# Double-Stranded RNAs (dsRNAs) as a Sustainable Tool against Gray Mold (*Botrytis cinerea*) in Grapevine: Effectiveness of Different Application Methods in an Open-Air Environment

**DOI:** 10.3390/biom10020200

**Published:** 2020-01-29

**Authors:** Luca Nerva, Marco Sandrini, Giorgio Gambino, Walter Chitarra

**Affiliations:** 1 Research Centre for Viticulture and Enology, Council for Agricultural Research and Economics (CREA-VE), Via XXVIII Aprile 26, 31015 Conegliano, Italy; 2 Institute for Sustainable Plant Protection, National Research Council (IPSP-CNR), Strada delle Cacce 73, 10135 Torino, Italy; 3 Dipartimento di Scienze Agroalimentari, Ambientali e Animali, Università degli Studi di Udine, Via delle Scienze 206, 33100 Udine, Italy

**Keywords:** dsRNA, spray induced gene silencing, plant protection, sustainability, grapevine, *Vitis vinifera*, *Botrytis cinerea*

## Abstract

Grapevine is one of the most important and globally widespread fruit species, with a high impact on the economy of many countries but with an intense environmental effect. Therefore, new environmentally friendly defense strategies against fungal pathogens are needed for more sustainable agriculture. A novel emerging approach is spray-induced gene silencing (SIGS), which concerns the exogenous application of double-stranded RNA (dsRNA) inducing enhanced plant resistance against fungal pathogens. Here, we tested the ability of SIGS to prevent and counteract infection of *Botrytis cinerea*, one of the most economically impacting pathogens of grapevine. In particular, we tested three independent approaches for dsRNA delivery into plants: (i) high pressure spraying of leaves; (ii) petiole adsorption of dsRNAs; (iii) postharvest spraying of bunches. We demonstrated that independently from the method of application, SIGS can reduce virulence of the fungus. Moreover, we also observed three different levels of efficacy depending on the method of application. Thus, the present data provide crucial information on the possibility to exploit SIGS as an alternative sustainable and ecofriendly strategy for grapevine pre- and postharvest protection.

## 1. Introduction

The global population is exponentially growing and, together with the ongoing climate changing scenario, is threatening food security [1]. One of the main issues is that more than 30% of the global agricultural production is lost in the field every year due to attacks from a multitude of pathogens [2], resulting in extensive use of pesticides and fungicides. Among all the different cultural systems, viticulture is considered one of the most treated cropping systems [3], with an intensive fungicide schedule used to control the spread of fungal pathogens (from 12 to 30 treatments per season) [4]. For these reasons, there is a need for new and more sustainable strategies to control pathogens. In line with this, modern agriculture (including viticulture) is now entering a new green revolution, thanks to gene function studies (both in plants and pathogens) and by the application of this knowledge in pest management strategies [5,6,7,8].

Among the most important fungal pathogens affecting grapevine, *Botrytis cinerea* (gray mold) is the cause of grape bunch rot, causing lower yields and reductions in fruit and wine quality [9]. The fungus becomes quiescent after infection of flower parts, only resuming growth when berries ripen, causing fruit rot [10]. In addition, *B. cinerea* shows saprophytic ability and nutritional versatility, giving it a survival advantage. During winter, it is able to survive in dead and infected plant tissues from the previous season, dormant or as saprophytically active mycelium [10,11]. In temperate climates, management of *B. cinerea* involves an integration of agricultural methods which create an unfavorable niche for fungal growth along with the application of fungicides. At least two factors can influence the application of antifungal compounds: (i) the use of fungicides close to harvest is limited by regulations to prevent residues in wines or in table grape; (ii) several cases of resistance to fungicides have been reported in *B. cinerea* [12].

One of the most intriguing processes involved in plant defense was discovered more than 20 years ago by Fire and Mello, who described the gene-silencing mechanism of eukaryotes, naming it RNA interference (RNAi) [13]. The system is based on the recognition of double-stranded RNA (dsRNA) molecules, which lead to the so called post-transcriptional gene silencing (PTGS) by degradation of the target messenger RNA (mRNA) [13]. Intriguingly, besides their role in endogenous gene regulation [14], small RNAs (sRNAs) are also involved in plant–pathogen interactions. For example, it is well known that once a virus enters a plant cell, viral-derived dsRNAs are targeted by the RNAi machinery to initiate a cascade of regulatory events which lead to the production of small interfering RNAs (siRNAs) [15]. In recent years, it has been reported that sRNAs can also act as effectors [16]: some pathogens and pests deliver sRNAs into plant cells to suppress host immunity [17] and, in parallel, plants can transfer sRNAs into pathogens and pests to lower their virulence [18]. To deepen the knowledge about sRNAs as effectors, the system model composed by *Arabidopsis thaliana* and *Botrytis cinerea* was studied [17]. By means of sRNAs sequencing, transgenic expression of fungal sRNAs, and pathogenicity tests, the authors demonstrated that *B. cinerea* produces several specific sRNA molecules able to shut down key genes of the plant immune system. This result was also confirmed by further experiments in which transgenic plants with impaired RNAi machinery displayed lower susceptibility, as well as mutant fungal strains for two proteins involved in sRNAs processing displaying lower virulence [17].

The possibility to exploit the silencing machinery for plant protection has arisen from a peculiar phenomenon called host-induced gene silencing (HIGS) [19]: transgenic plants expressing artificial dsRNAs can induce gene silencing in interacting pests and pathogens, delivering to them siRNAs, which act as effectors against the invader [20,21,22,23,24]. Despite the high potentiality of this technique, the main factors limiting its utilization are the lack of transformation protocols for many crop species and regulatory restrictions for genetically modified (GM) plants [25]. In Europe, the use of transgenic plants is strictly normed, and several countries are still denying the possibility of their use due to ethical concerns (the so called ‘GMO opt-out’). To overcome these problems, dsRNAs can be directly applied on leaves or fruits, offering shorter-term plant protection through an alternative RNAi strategy. Recent works have demonstrated that the RNA spraying method, called spray-induced gene silencing (SIGS), is effective in controlling fungal pathogens [26,27]. In specific, Wang and colleagues [27] showed that exogenously applied fluorescein-tagged dsRNAs are absorbed by fungal mycelia in about 12 h, leading to reduced accumulation of target mRNAs when compared to the untreated control. Similarly, when dsRNAs were applied on fruits, leaves, or flowers of several species, they observed reduced fungal virulence.

When grapevine fruits are detached from plants and until cell death occurs, they remain metabolically active, reacting to external stresses by changing their metabolism and, as a consequence, modifying their phytochemical composition [28]. For this reason, postharvest strategies are generally aimed at reducing metabolic activity to preserve the original physicochemical properties [29]. For some special foods, postharvest controlled stresses to induce specific metabolic changes are desired, such as for grape, where water loss is needed for raisin production or for making special wines [30]. As previously mentioned, *B. cinerea* is one of the main pathogens threatening grape during ripening and postharvest, limiting its shelf-life for both the table grape and wine making industry. In light of this, we report the possibility to control pre- and postharvest gray mold infections by using SIGS, an environmental friendly and sustainable alternative to fungicides.

## 2. Materials and Methods

### 2.1. Plant Material

The experimental trial was carried out at the beginning of September 2019 at CREA—Research Centre for Viticulture and Enology (45°51′10.1″ N, 12°15′21.2″ E), working on seven-year old potted grapevines (*Vitis vinifera* L.) using the *B. cinerea*-susceptible cultivar Moscato [31,32] (white grape variety) grafted onto Kober 5BB rootstock and maintained in an open air environment. Plants were grown in 150 L pots using a peat substrate (TS4, Turco Silvestro, Italy) and placed over a woven polypropylene geotextile mulching film under a greenhouse shade cloth in order to protect them from leaf- and fruit-damaging hailstorms. Fungal material used in this study corresponded to an isolate of *B. cinerea* collected from naturally infected grapevines cv. Moscato grown in a nearby experimental vineyard and maintained under in vitro conditions to obtain conidia.

### 2.2. dsRNAs—Design and Production

Three essential *B. cinerea* genes were selected considering the fungicide site of action (see Results and Discussion section): *BcCYP51*, *Bcchs1*, and *BcEF2*. To amplify a fragment of the fungal essential genes, specific primers were developed on non-conserved regions. Primers were designed to be suitable for overlapping PCR in order to obtain a final PCR amplicon with all the selected sequences (Table S1). The PCR product was cleaned using the DNA Clean & Concentrator Kit (Zymo Research, CA, USA) following manufacturer instructions. Specific restriction enzyme digestion was then performed with PstI (Thermo Fisher Scientifc, MA, USA) to clone the PCR amplicon into plasmid L4440, which contains a double and convergent T7 promoter to produce dsRNA (Addgene plasmid #1654) [33]. L4440 was previously linearized with PstI and dephosphorylated with alkaline phosphatase (Thermo Fisher Scientific, MA, USA) to prevent self-ligation. Ligation products were transformed into chemically competent DH5α *E. coli* cells. In order to confirm the correctness of plasmid sequences, positive colonies were grown overnight in 4 mL of LB plus antibiotic, subjected to plasmid extraction using the Zyppy™ Plasmid Miniprep Kit (Zymo Research, Irvine, CA, USA), and sequenced with Sanger method [34] at Bio-Fab Research (Roma, Italy) as previously mentioned [35].

A similar approach was used to obtain dsRNAs with GFP sequence. Specific primers were designed to amplify a 376 bp amplicon from the pCBCT plasmid (Table S1). Both forward and reverse primers contain a PstI restriction site in order to allow cloning into linearized L4440 plasmid. Ligation, transformation of DH5α *E. coli* cells, and plasmid production were performed as above mentioned.

The plasmid carrying the custom-built sequence was transformed into HT115 (DE3) *E. coli* chemically competent cells [36]. Bacterial cells were plated on LB media supplemented with 12.5 µg/mL of tetracycline and 100 µg/mL of ampicillin. Positive colonies were then screened by PCR [37] and stored at −80 °C for the subsequent operation.

To produce dsRNAs, a single colony carrying the selected plasmid was grown in 10 mL of LB amended with antibiotics overnight. The following day, 1 L of LB with antibiotics was inoculated with the 10 mL of bacterial culture and shaken at 37 °C, 215 rpm, for 4 h. Then, IPTG was added to a final concentration of 400 µM and bacteria were left to grow for an additional 4 h. Bacteria cells were then pelleted and stored at −80 °C until further processing.

Extraction of dsRNAs was achieved through a classic phenol–chloroform extraction followed by isopropanol precipitation [38]. RNA obtained was treated with DNaseI (Thermo Fisher Scientifc, Waltham, MA, USA) and dsRNA integrity was checked with a 1% agarose gel. To quantify the amount of dsRNA, a weighted ladder was used (HyperLadder™ Molecular Weight Markers, Bioline, UK) (Supplementary Figure S1).

### 2.3. Effects of In Vitro and In Vivo dsRNA Delivery on B. cinerea Development

The effects of produced dsRNAs on *B. cinerea* growth and virulence were first tested in vitro using potato dextrose agar (PDA, HiMedia Laboratories, The Netherlands) plates. Plugs 8 mm in diameter were obtained from a *Botrytis* colony actively growing for 6 days. One plug was placed at the center of each plate and then, for each treatment, 500 ng (30 µL) of dsRNA was immediately added on top of the plug and then continuously supplied every 12 h. Three conditions were tested: (i) dsRNA carrying *B. cinerea* sequences (*Bc* dsRNA); (ii) dsRNA carrying GFP sequence (GFP dsRNA); (iii) water as control treatment (H_2_O). Growth dynamics was evaluated by measuring the diameter of colonies at 1, 2, 3, and 5 days post-inoculation.

We then evaluated the in vivo effects by means of fungal development on grape bunches. Experiments were carried out using three application methods (plus an untreated control) on two independent blocks of plants for each treatment (3 plants × 3 application method × 4 treatments × 2 blocks = 72 plants in total). In detail, in addition to each condition reported above (*Bc* dsRNA; GFP dsRNA; H_2_O) a control condition (CTRL) was added. The three application methods selected were (i) high pressure spraying on plant leaves avoiding bunches (about 300 µg in 3 mL of ddH_2_O per plants of dsRNAs); (ii) leaf petioles adsorption (about 300 µg in 3 mL of ddH_2_O per plants of dsRNAs); (iii) high pressure spraying on postharvest berry bunches (about 10 µg in 500 µL of ddH_2_O per bunch of dsRNAs).

**Figure 1:**
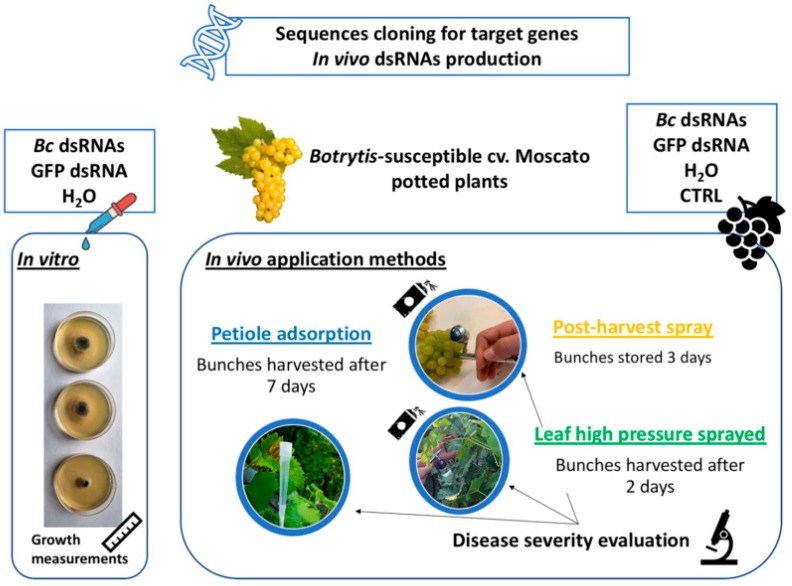
Experimental workflow overview of in vitro and in vivo experiments.

From each plant, three randomly selected bunches were harvested and inoculated with pathogen (*Bc* +,1 mL per bunch, 10^5^ conidia per mL) and one was harvested and maintained as uninoculated control (*Bc* −). Then, each bunch was kept in an independent plastic box with wet filter paper (3 mL per box) placed on the bottom to retain high humidity and induce pathogen development. In detail, four bunches were harvested per plant for each condition and application method: for SIGS we harvested bunches 2 days after dsRNAs application, while in the petiole adsorption method, we harvested bunches 7 days after dsRNAs inoculation [39]. Pathogen was inoculated (1 mL per bunch, 10^5^ conidia per mL) at the time of harvesting for both leaf and petiole application methods. Finally, for postharvest trials, bunches were harvested and stored at room temperature for 3 days, then dsRNAs were applied and fungal pathogen inoculated 2 days after. For each conditions and application method, ten days after pathogen inoculation disease development was evaluated as percentage of berries attacked in every bunch [32,40]. An experimental overview is provided in Figure 1.

Significant differences among samples were analyzed by one-way ANOVA test, using the Tukey’s HSD post hoc test for separating means when ANOVA results were significant (*p < 0.05*).

### 2.4. RNA Extraction and Northern Blot Analysis

Distal leaf and berry samples from petiole adsorption method were harvested for checking dsRNA mobility and accumulation over time at 3 and 7 days post dsRNA inoculation. RNA was extracted using the Spectrum plant total RNA kit (Merck KGaA, Darmstadt, Germany) following the manufacturer’s instructions. Northern blotting was carried out according to a previously published method [41]. Briefly, 2 μg of total RNA was separated by electrophoresis on a 1.2% denaturing formaldehyde agarose gel and capillary-blotted onto a nylon membrane positively charged. The *Bc* dsRNA and grapevine 18S rRNA were used as probes by digoxigenin labeling with a PCR DIG Probe Synthesis Kit (Merck KGaA, Darmstadt, Germany) according to the manufacturer’s instructions. Hybridizations, Dig chemiluminescent detection, and probe stripping procedures were carried out following manufacturer’s instructions (Merck KGaA, Darmstadt, Germany).

## 3. Results and Discussion

To provide a proof of concept and show the potentiality of RNAi technology against pre- and postharvest grape berry pathogens, we conducted an experiment targeting three independent essential genes of *B. cinerea* which were selected on the basis of fungicide sites of action found in literature [42,43]. The first selected gene was lanosterol 14α-demethylase (*erg11* or *CYP51*), which is the primary target of triazole antifungal agents. This class of compound was one of the most efficient, widely used for fungal control, for which we are now observing severe drug resistance also for human pathogens [44]. The *erg11* gene belongs to the cytochrome P450 monooxygenase (CYP) superfamily and mediates a crucial step in the biosynthesis of ergosterol, which is a fungal-specific sterol [45]. Since chitin, in fungal cell walls, is unique to fungi, it provides ideal targets for the development of selective antifungal agents and, for this reason, we selected chitin synthase 1 (*chs1*) as the second target for our RNAi protocol. The gene is involved in polymerization of N-acetylglucosamine from UDP-N-acetylglucosamine and plays a crucial role in cell wall polymerization [46]. It has been already demonstrated that the inactivation of *chs1* in *B. cinerea* leads to stunted growth and a weakened cell wall structure [47]. Finally, we identified elongation factor 2 (*EF2*) as the third essential gene. The latter catalyzes ribosomal translocation during protein synthesis and is a target for sordarin-derived antifungal compounds [48]. Once the three targets were identified, we proceeded by amplifying a small fragment (about 200–250 bp) of each with custom designed primers. Primers were designed with adaptors to allow overlapping PCR and produce a final PCR amplicon of 732 bp containing sequences from the three different genes. A PCR amplicon of 385 bp was obtained from GFP sequence in the pCBCT plasmid and further used as control for dsRNAs inoculation. For both *Bc* dsRNAs and GFP dsRNAs, we were able to produce up to 2 mg of dsRNA for each liter of bacterial culture.

**Figure 2:**
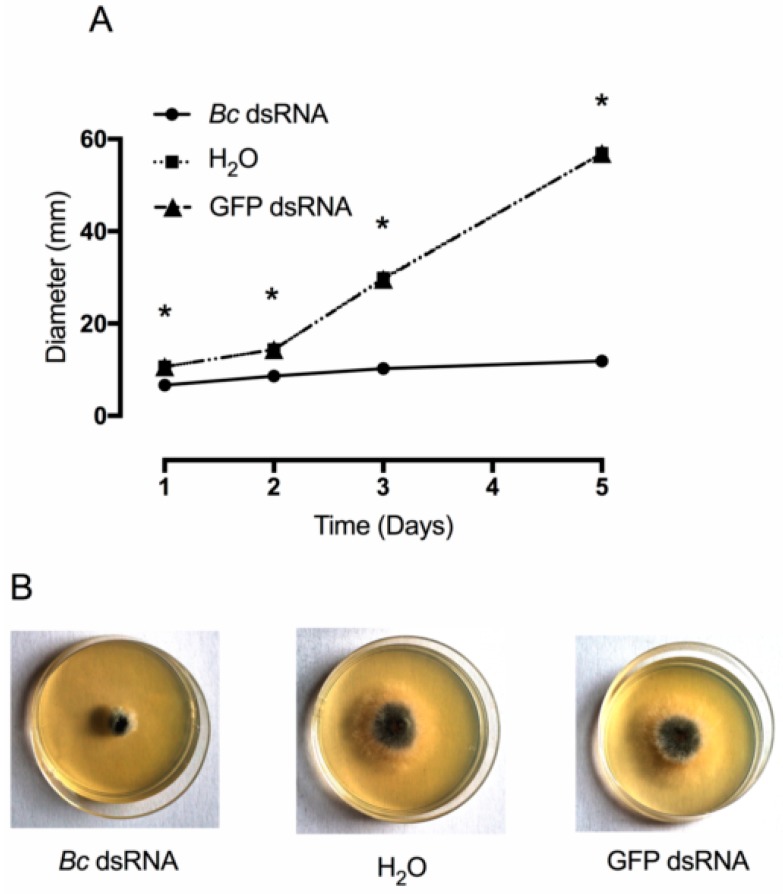
Effect of supplied dsRNAs in vitro. Growth of *B. cinerea* over time following the application of *Bc* dsRNA, GFP dsRNA, and H_2_O (**A**). Examples of fungal development in petri dishes in response to dsRNAs or H_2_O delivery at three days (**B**). Asterisks indicate significant differences as determined by Tukey HSD test (*p* ≤ 0.05).

After dsRNAs production, we proceeded to test their ability to reduce growth of *B. cinera* in axenic conditions. As is shown in Figure 2A,B, colonies exposed to *Bc* dsRNA showed a significant reduction in growth compared to colonies exposed to GFP dsRNAs or water. Interestingly, during a preliminary trial, we supplied *Bc* dsRNAs only at the time of plate inoculation, observing a reduction in mycelia growth in the first 24 h, but not at later times. For this reason, we repeated the experiment supplying dsRNAs (or water in the case of negative control) every 12 h. Probably, as also observed for *Fusarium asiaticum*, the ability of *B. cinerea* to maintain secondary amplification of the RNAi signal could be impaired [49]. It is well known that two distinct types of sRNAs participate in RNAi: primary siRNAs which are derived from dicer nuclease-mediated cleavage and secondary siRNAs whose synthesis requires an RNA-dependent RNA polymerase (RdRP) [50]. Although the core RNAi machinery appears to be widely conserved in fungi [51], it is also known that many exceptions exist [52]. For example, some fungi show dicer-independent pathway or some others have completely lost the RNAi machinery along evolution [53]. In the light of this, we can speculate that the isolate of *B. cinerea* used in the present study is impaired in the amplification of secondary siRNA, but further studies are needed to confirm this result.

**Figure 3:**
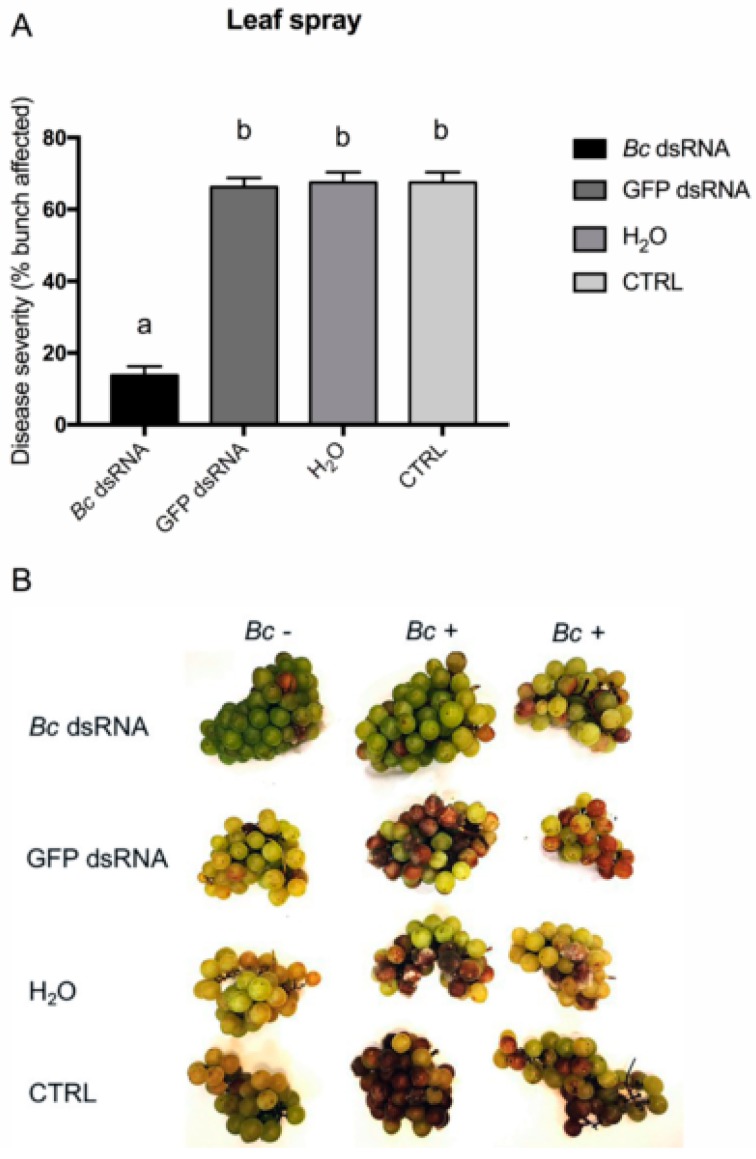
Disease severity on bunches recorded for the in vivo high pressure leaf spray application method. Percentage of berries affected following *Bc* dsRNA, GFP dsRNA, H_2_O application, and in untreated CTRL samples. Different lowercase letters above bars indicate significant differences as determined by Tukey HSD test (*p* ≤ 0.05) (**A**). Overview of representative samples for each condition tested. One bunch was maintained as uninoculated control (*Bc* −) and two inoculated (*Bc* +) bunches of cv. Moscato are shown (**B**).

Since the axenic culture gave interesting results, we decided to move our experiments onto grapevine plants, testing the ability of produced molecules to impair fungal growth and virulence on berry bunches which is the most economically impacting site of infection. Three different effective methods for dsRNA application have been reported in the literature: the first is the application onto leaf by high pressure spraying. This specific approach allows dsRNAs to enter into plant cells, which will then process them into siRNAs [54]. One of the features of plant RNAi is the ability to amplify the initial response through RdRPs which, as already mentioned, synthesize secondary siRNAs [55]. Secondary siRNAs are more abundant than primary siRNAs and display a stronger impact on target gene expression level [56]. In our experiments, application of dsRNAs through high pressure spraying leads to a significant reduction in pathogen development only when *Bc* dsRNAs were applied, as shown in Figure 3A,B. The observed results confirmed the efficacy of the selected sequences against *B. cinerea*.

**Figure 4:**
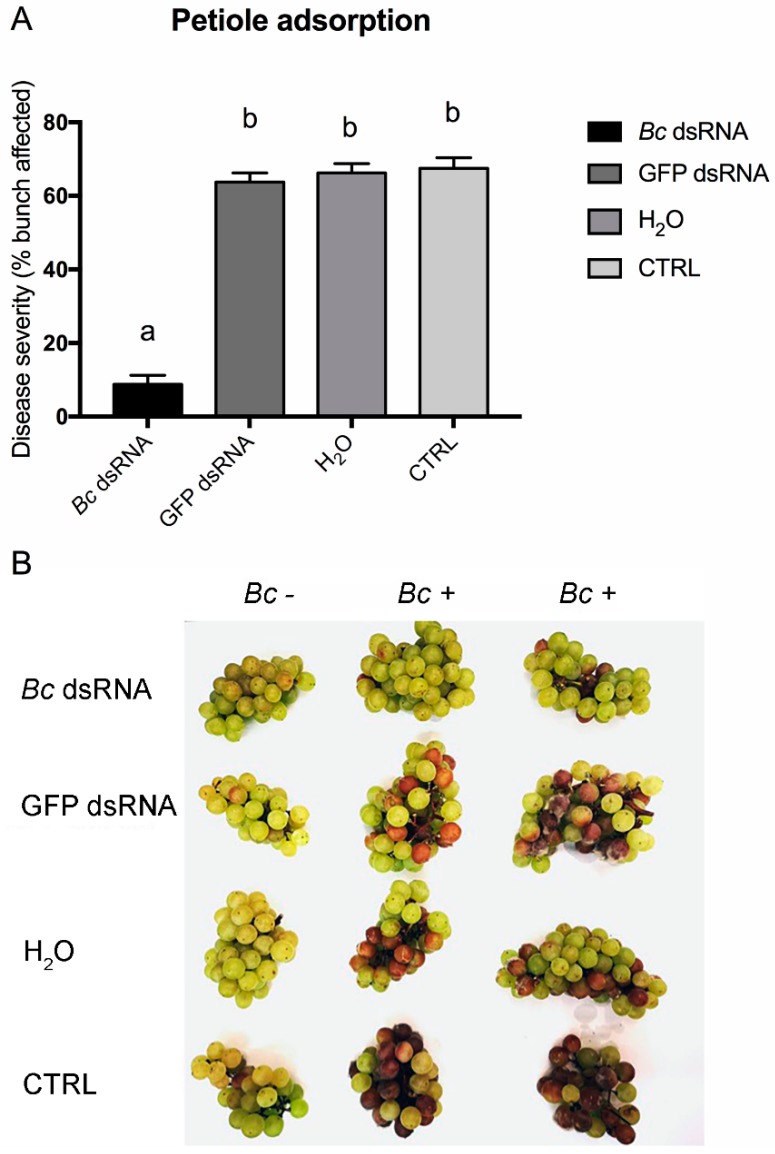
Disease severity on bunches recorded for the in vivo petiole adsorption application method. Percentage of berries affected following *Bc* dsRNA, GFP dsRNA, H_2_O application and in untreated CTRL samples. Different lowercase letters above bars indicate significant differences as determined by Tukey HSD test (*p* ≤ 0.05) (**A**). Overview of representative samples for each condition tested. One bunch was maintained as uninoculated control (*Bc* −) and two inoculated (*Bc* +) bunches of cv. Moscato are shown (**B**).

A second method of application was based on dsRNAs delivery through petiole adsorption (Supplementary Figure S2). It was demonstrated that if a petiole from a detached leaf is immediately put in contact with a liquid containing dsRNAs, it can work as source, adsorbing the liquid and leading to the upstream transportation of dissolved molecules [39]. The interesting feature of this method is that the adsorbed liquid moves into xylem vessels and into the apoplastic space, leading to the spread of intact dsRNAs inside the plant [39]. To deliver solutions into open-air growing vines, we developed a system using 5 mL tips (in which the solution was loaded) linked immediately after leaf detachment in order to avoid embolism formation in xylem vessels (Supplementary Figure S2). In addition, as already observed in another work [39], the diffusion level of dsRNAs into plant tissues increased over time, reaching the maximum at 7 days post application, as demonstrated by Northern blot analysis (Supplementary Figure S3). Disease development scoring showed that only *Bc* dsRNA-treated plants had less susceptible bunches, as shown in Figure 4A,B. Moreover, as shown in Supplementary Figure S3, dsRNAs seem to accumulate more in berries than in leaves. This is probably due to the time of treatment, in September, when the ripening processes are still ongoing and berries transpire a considerable amount of water to regulate temperature and osmotic pressure [57].

**Figure 5:**
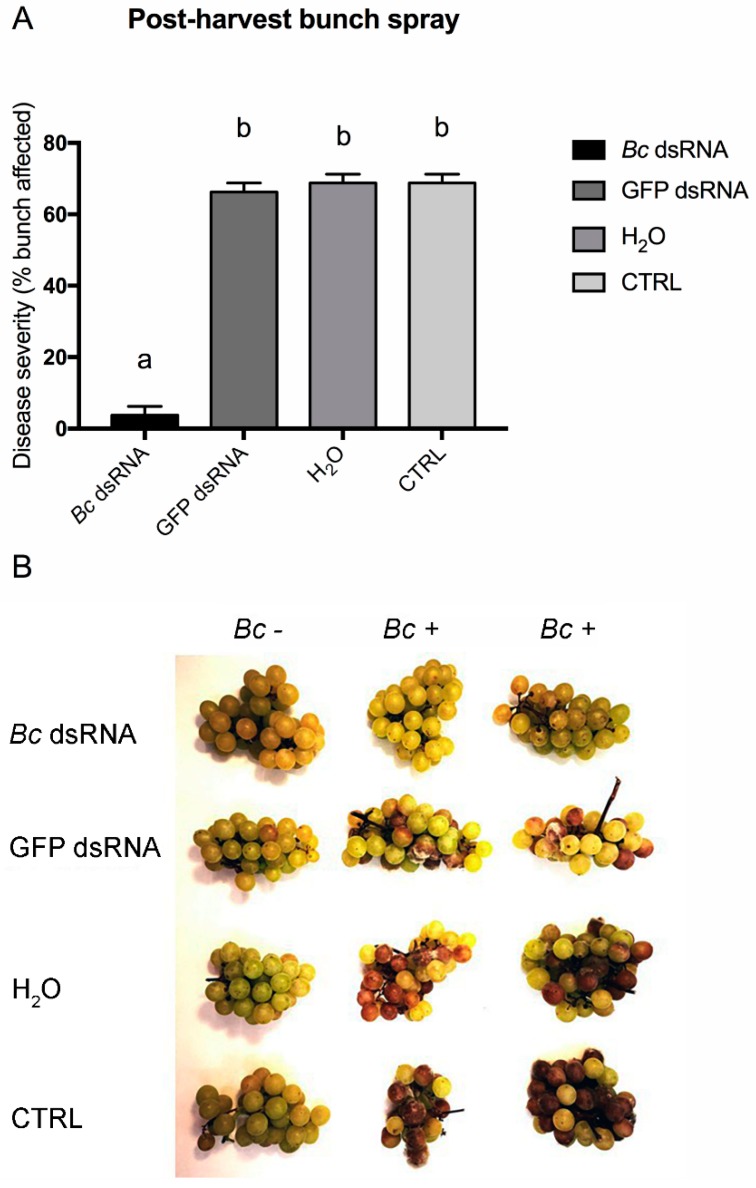
Disease severity on bunches recorded for the in vivo high pressure spray application method in postharvest bunches. Percentage of berries affected following *Bc* dsRNA, GFP dsRNA, H_2_O application and in untreated CTRL samples. Different lowercase letters above bars indicate significant differences as determined by Tukey HSD test (*p* ≤ 0.05) (**A**). Overview of representative samples for each condition tested. One bunch was maintained as uninoculated control (*Bc* -) and two inoculated (*Bc* +) bunches of cv. Moscato are shown (**B**).

Finally, the third application method tested was the high pressure nebulization of dsRNAs solutions directly on harvested grape bunches. As was already demonstrated, application of dsRNAs on leaf surfaces inhibits fungal growth in both the directly sprayed as well as the distal non-treated parts [26]. Moreover, the same authors demonstrated that control of fungal infections in non-treated tissues involve adsorption and movement through the plant vascular system of intact dsRNAs. In our experiment, the application of dsRNAs on berry surfaces resulted in reduced disease development, as shown in Figure 5A,B.

**Figure 6:**
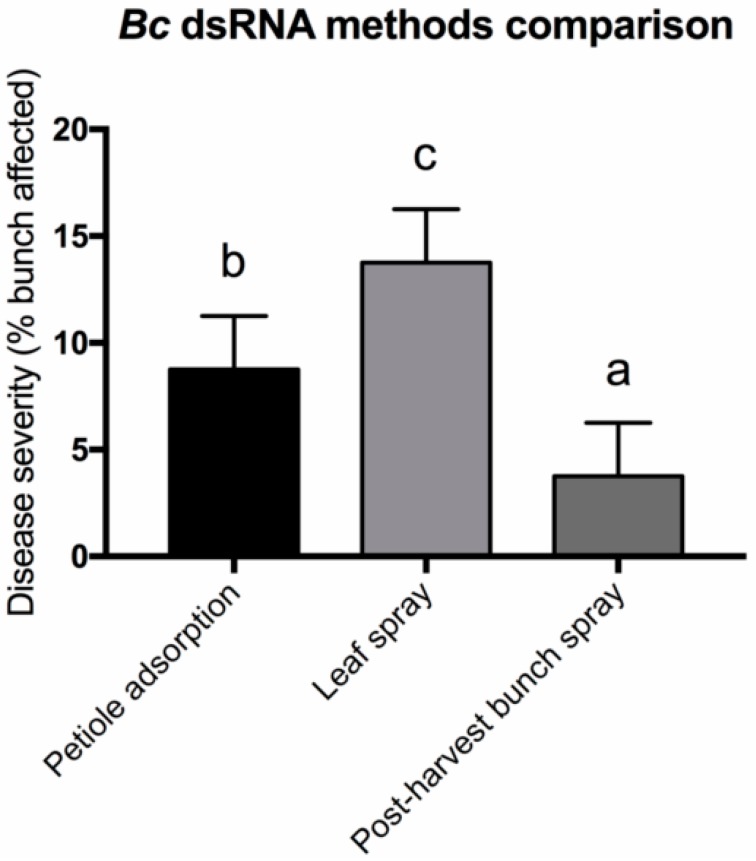
Comparison of disease severities among application methods used in this study using *Bc* dsRNAs. Different lowercase letters above bars indicate significant differences as determined by Tukey HSD test (*p* ≤ 0.05).

To determine which of the three methods could represent the best approach to control *B. cinerea* infections, we compared the disease development of *Bc* dsRNA-treated bunches. As reported in Figure 6, we observed three different levels of efficacy: the most effective application method was the high pressure spraying of dsRNAs on berry bunches in postharvest grapes. The second most effective method was through petiole adsorption and, finally, the least effective was high pressure leaf spraying, which showed a disease severity more than double if compared with the postharvest application. Despite the lower amount of dsRNA that was applied in the first method compared to the other two approaches, we observed a higher efficiency in controlling pathogen development. We cannot exclude that application through petiole adsorption and high pressure spraying in leaves led to the different accumulation of dsRNA molecules in bunches. For this reason, further investigations are needed to confirm this result. Moreover, despite in vitro application, petiole adsorption and postharvest methods provide intact dsRNAs to the fungus, and only in vivo applications do not require a continuous dsRNAs supply, displaying long-lasting effectiveness. This is probably due to the fact that sprayed dsRNAs are adsorbed by plant tissues and are not directly available for fungal uptake [26]. Hence, it is conceivable that the dsRNAs are continuously supplied to the fungus over its growth on plant tissues through slow plant-mediated movement and release in the xylem and apoplast compartments.

Furthermore, the two most effective application methods used in this study were those in which intact dsRNAs are provided to the fungus. A possible explanation for this phenomenon is linked to the specificity of fungal RNAi machinery [53]. It has been demonstrated that fungal sRNA patterns differ from that of plants: the typical plant siRNA sizes are conserved and range between 21–22 and 24 nucleotides in length [58,59,60]. In fungi, the siRNA lengths range from 21 up to 30 nucleotides in length, also within the same fungal family [38,61,62]. In light of this, we can speculate that argonaute proteins (AGO) and dicer-like proteins (DCL) of fungi can better recognize intact dsRNAs with respect to plant siRNAs that are already processed.

In this study, we demonstrated that dsRNAs can confer protection against *B. cinerea* in both pre- and postharvest conditions. In addition, this is the first trial in which several application methods were compared in a natural environment. To date, the majority of trials were performed only in vitro or on small plant explants [26,27,63], but we demonstrated the ability of SIGS to be implemented in real conditions. The potentiality of SIGS, as an alternative method to conventional chemicals, represents a challenging opportunity for researchers and agricultural-related industries. SIGS show many advantages: (i) specificity can be managed by choosing a more or a less conserved nucleotide sequence; (ii) it is possible to develop specific sequences for an unlimited range of pathogens, with the only request being an active RNAi machinery. Moreover, while HIGS has been frequently demonstrated to be easily achievable [23,64,65,66], only a few examples have demonstrated the efficiency of exogenous RNA delivery to kill fungal pathogens [26,63,67], as observed in our study. Although the exact mechanism of external RNA recognition, uptake, and transport remains to be determined, the potentiality of SIGS as sustainable strategy for grape and plant protection is becoming more and more real. In the future, commercial applications of SIGS will be conceivable, although some problems still need to be fixed: (i) cost-efficient dsRNAs production at large scales must be achieved, (ii) specific application tools need to be developed, as well as (iii) stabilizing agents useful for field conditions. Therefore, more research into practical issues will be required to exploit this promising technique in sustainable viticulture and, more generally, the context of agricultural defense management.

## Supplementary Materials

The following are available online at https://www.mdpi.com/2218-273X/10/2/200/s1, Table S1: List of primer, Figure S1: Agarose gel electrophoretic analysis of dsRNAs, Figure S2: Overview of in vivo petiole adsorption, Figure S3: Northern blot analysis.
