# Supplementary material for: Double-Stranded RNAs (dsRNAs) as a Sustainable Tool against Gray Mold (Botrytis cinerea) in Grapevine: Effectiveness of Different Application Methods in an Open-Air Environment
Source: Biomolecules. 2020 Jan 29;10(2):200. doi: 10.3390/biom10020200 (PMC7072719; doi:10.3390/biom10020200)

Supplementary files

Double-Stranded RNAs (dsRNAs) as a Sustainable Tool against Gray Mold (Botrytis cinerea) in Grapevine: Effectiveness of Different Application Methods in an Open-Air Environment

Luca Nerva[1,2,*], Marco Sandrini[1,3], Giorgio Gambino[2], Walter Chitarra[1,2,*]

[1]Research Centre for Viticulture and Enology, Council for Agricultural Research and Economics (CREA-VE), Via XXVIII Aprile 26, 31015 Conegliano (Italy)

[2]Institute for Sustainable Plant Protection, National Research Council (IPSP-CNR), Strada delle Cacce 73, 10135 Torino (Italy)

[3]Dipartimento di Scienze Agroalimentari, Ambientali e Animali, Università degli Studi di Udine, Via delle Scienze 206, 33100 Udine (Italy)

***Corresponding authors**

Luca Nerva and Walter Chitarra

Research Centre for Viticulture and Enology, Council for Agricultural Research and Economics (CREA-VE), Via XVIII Aprile 26, 31015 Conegliano (Italy)

Institute for Sustainable Plant Protection, National Research Council (IPSP-CNR), Strada delle Cacce 73, 10135 Torino (Italy)

Tel: +390438456712

Fax: +390438450773

e-mail: walter.chitarra@crea.gov.it

luca.nerva@crea.gov.it

Supplementary Table 1. List of primer designed to be used in overlapping PCR.

Target Gene	NCBI Accession	Primer name	Primer sequence
3-ketosteroid reductase (erg27)	JX436969.1	Bc_3Keto_F	AAAACTGCAGGATACGGTGGCTCGGGTTCA
		Bc_3Keto_R	TTGAGGGTGCGGCGATTGATCTCGCCTTCAGACCCTTCCT
Chitin syntase 1 (CHS1)	XM_024694912.1	Bc_CHS1_F	AGGAAGGGTCTGAAGGCGAGATCAATCGCCGCACCCTCAA
		Bc_CHS1_R	TGCCATACTCGACACCCTCCTGGGATTGAGGGGCATGC
Elongation factor 2 (ef2)	XM_024693463.1	Bc_EF2_F	GCATGCCCCTCAATCCCAGGAGGGTGTCGAGTATGGCA
		Bc_EF2_R	AAAACTGCAGGCGGGGAGGAAGTCTGTGAC
Green fluorescent protein (GFP)	From pCBCT plasmid	GFP_F	AAAACTGCAGACCCTGAAGTTCATCTGCACCAC
		GFP_R	AAAACTGCAGGATCTTGAAGTTGACCTTGATGCC

Supplementary Figure 1. Agarose gel electrophoretic analysis of *Bc* dsRNA fragments after DNAse treatment. Samples were loaded as 0.5-1-5 μL of total nucleic acid isolation as detailed in Materials and Methods section. WL: weighed ladder; M: size molecular marker.

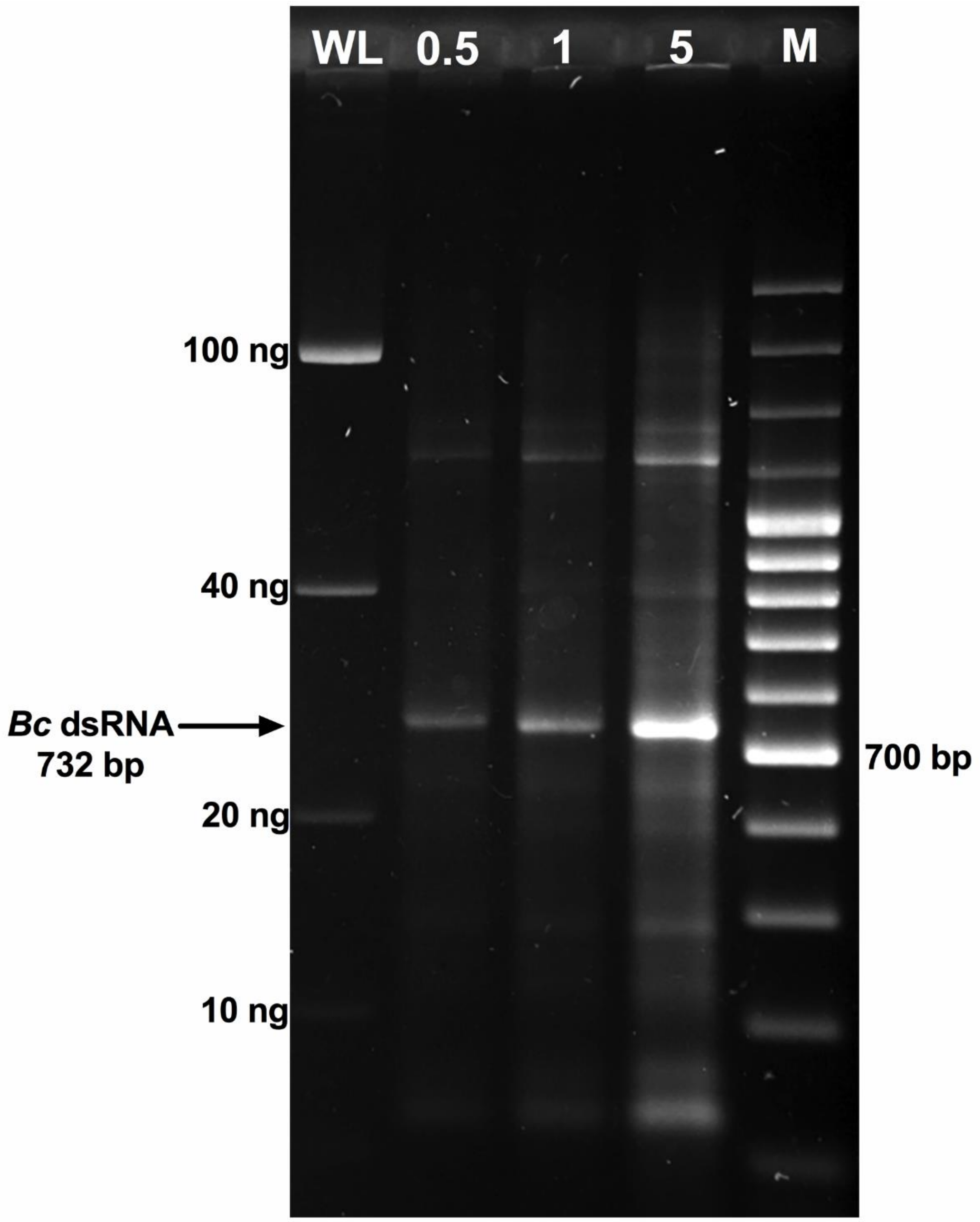

Supplementary Figure 2. Overview of *in vivo* petiole adsorption application method.

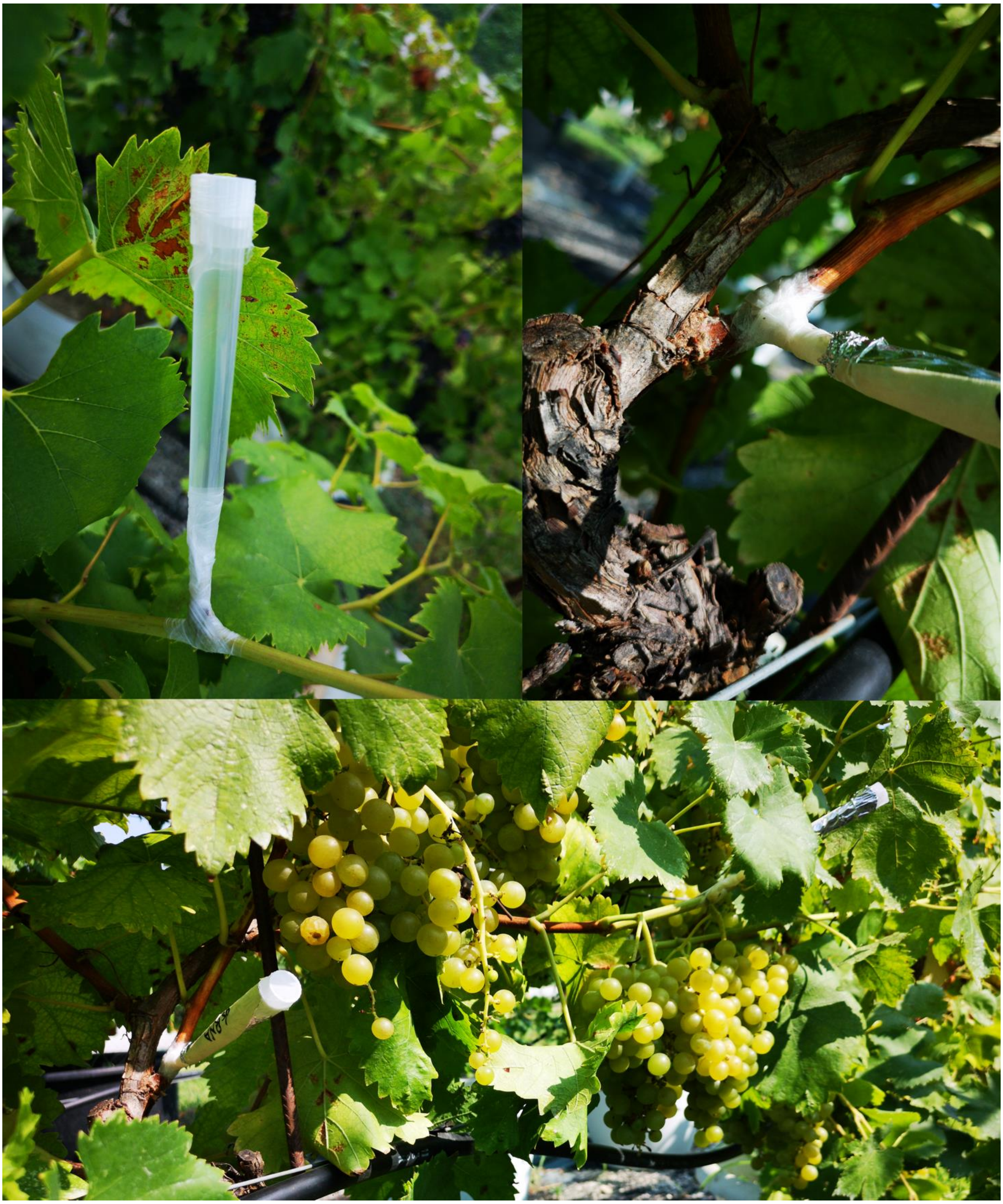

Supplementary Figure 3. Northern blot analysis of *Bc* dsRNA in Leaf and Berry tissues over time. '–' indicate untreated samples; '+' indicate *Bc* dsRNA treated samples. ds+plant: *Bc* dsRNA added to an untreated leaf sample used as positive control.

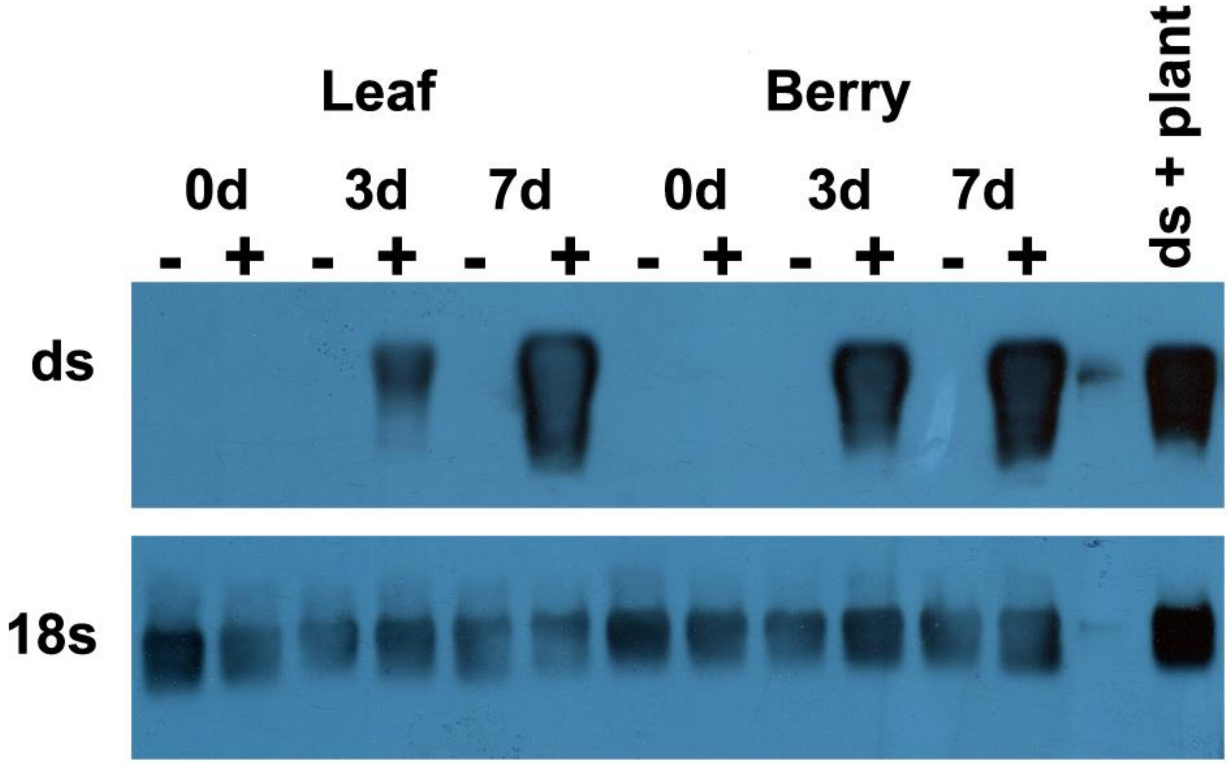

Supplement: Supplementary file 1 [file biomolecules-10-00200-s001.pdf]
